# Assessing the quantumness of the annealing dynamics via Leggett Garg’s inequalities: a weak measurement approach

**DOI:** 10.1038/s41598-019-50081-8

**Published:** 2019-09-20

**Authors:** V. Vitale, G. De Filippis, A. de Candia, A. Tagliacozzo, V. Cataudella, P. Lucignano

**Affiliations:** 1 Dipartimento di Fisica “Ettore Pancini”, Università di Napoli “Federico II”, Monte S. Angelo, I-80126 Napoli Italy; 2 CNR-SPIN, Monte S. Angelo via Cinthia, I-80126 Napoli Italy

**Keywords:** Condensed-matter physics, Quantum physics

## Abstract

Adiabatic quantum computation (AQC) is a promising counterpart of universal quantum computation, based on the key concept of quantum annealing (QA). QA is claimed to be at the basis of commercial quantum computers and benefits from the fact that the detrimental role of decoherence and dephasing seems to have poor impact on the annealing towards the ground state. While many papers show interesting optimization results with a sizable number of qubits, a clear evidence of a full quantum coherent behavior during the whole annealing procedure is still lacking. In this paper we show that quantum non-demolition (weak) measurements of Leggett Garg inequalities can be used to efficiently assess the quantumness of the QA procedure. Numerical simulations based on a weak coupling Lindblad approach are compared with classical Langevin simulations to support our statements.

## Introduction

Although any quantum algorithm can be run on adiabatic quantum computers^1^, the interest of the scientific community is focused on decision and optimization problems that are very difficult to handle on classical computers, because their computational time, most of the times, grows exponentially with the number of bits. Optimization problems can be mapped onto complex many body hamiltonians^2^, hence AQC is also of outmost interest from the fundamental point of view, as it may provide insights into longstanding problems in modern condensed matter physics such as physics of the strongly correlated cuprate materials^3^ and of the spin glasses^4^. AQC^5^ is founded on QA^6^, a slow quantum dynamics that proceeds from an initial Hamiltonian with a trivial ground state (easy to prepare), to a final Hamiltonian whose ground state encodes the solution of the computational problem. The adiabatic theorem guarantees that the system will track the instantaneous ground state if the Hamiltonian varies sufficiently slowly^7^. QA can perform better than thermal annealing^8,9^, however, to the date, there are only a few problems where this quantum speed-up has been clearly demonstrated.

Furthermore, in the last few years, there has been a renewed interest in QA^10–14^, because physical superconducting implementations of “quantum” annealers^15–17^ up to thousand of spins have been used to obtain the GS of interacting many body Hamiltonians. Moreover also optical techniques have been successfully used^18,19^, though with a smaller number of qubits. In real devices, the presence of the environment introduces decoherence and thermalization time scales that have to be longer than the annealing time^20^. This condition does not strictly guarantee that the dynamics can be assumed to be quantum for the whole evolution. Moreover, as the presence of coupling to an environment is unavoidable, the questions we want to address in the present study are the following:

(i) to what extent, the QA can be considered a quantum coherent dynamics, in the presence of a dissipative environment?
(ii) once we choose an annealing time *t*_*f*_ at which the system has evolved close to the target state, in a way to be defined below, is it possible to find an estimator of the “quantumness” of the evolution towards such state?

Giving a clear answer to the previous questions, is not only a matter of semantics. It is well known that quantum speed up^21^ can only be accessed if quantum mechanical coherence is preserved during the whole dynamics.

In order to answer to these questions, we propose to evaluate the Leggett-Garg’s inequalities (LGI) during the QA. LGI were developed in 1985, to study quantum coherence properties of macroscopic quantum systems^22^, that have been recently studied to assess the quantumness of a damped two level system^23^. They are Bell’s-like inequalities in time, and predict anomalous values for some correlation functions that are only possible if the system behaves according to quantum mechanics. They also provide sharp bounds for classical correlation functions^24^.

In this paper we focus on a simple model i.e. we study the QA of a single qubit and of two entangled qubits, in which measurements are performed just on one of the two.

The evolution of the single qubit in presence of a dissipative environment is studied as a test, although the quantum dynamics of a single qubit does not contain additional relevant features of an interacting multi-qubit system. Phenomena like many-body tunnelling, entanglement, and many body localization, do not show up in the case of a single qubit, of course. However, the study of the evolution of a single qubit is quite useful, as a benchmark for the two qubit case. It allows to characterize the degree of adiabaticity and to set up a simulated weak measurement process, checked to be as less invasive as possible for given time scales involved in the process. Explicit numerical simulations for the single/double qubit case, are the fundamental first step for understanding decoherence and relaxation phenomena out of equilibrium. In our simulations, we will monitor how quantum mechanical coherent behaviors are spoiled by the system-bath interaction and will discuss the optimization of the “external” parameters. As we will show, our approach can be easily generalized to the multi-qubits case, provided just one of them is acted on by the measuring apparatus.

## Model Hamiltonian for the single qubit case and its dissipative evolution

Let us consider the time evolution of a single qubit, up to time *t*_*f*_, so-called annealing time. ${\sigma }_{i}(i=x,z)$ are the Pauli spin 1/2 matrices describing the qubit, mimicked as a quantum two level system. At the initial time $s=0$ ($s=t/{t}_{f}$, $s\in [0,1]$), the system is prepared into the GS that is the *σ*_*x*_ eigenstate $|GS(0)\rangle =1/\sqrt{2}(|\,\uparrow \,\rangle -|\,\downarrow \,\rangle )$, and it is eventually annealed at the final time $s=1$ towards $|GS(1)\rangle =|\,\downarrow \,\rangle$. The QA is described by the following time-dependent Hamiltonian:

1 $$H(s)=(1-s)\frac{{\Gamma }_{x}}{2}{\sigma }_{x}+s\frac{{\Gamma }_{z}}{2}{\sigma }_{z}.$$

Following ref.^25^, we set our time/energy scale choosing ${\Gamma }_{x}={\Gamma }_{z}=1\,{\rm{GHz}}$ (that is the typical working frequency of the experimentally relevant annealers based on superconducting flux qubits^15^) and express all the energies in units of ${\Gamma }_{x}$ (times in units of $1/{\Gamma }_{x}$, $\hslash =1$). The qubit environment is described by a bath of bosonic harmonic oscillators in thermal equilibrium at the inverse temperature $\beta =1/{k}_{B}T$ (*k*_*B*_ is the Boltzmann constant). The qubit-bath coupling is described by an ohmic spectral density whose effective interaction strength is the dimensionless parameter *α*. At $\alpha =1$ (for $s=0$) the system undergoes the Leggett transition^26^, however in this paper we will focus on the weak coupling limit ($\alpha \ll 1$) far away from this critical point and adopt the Lindblad approach (for details see refs^20,27^). The reduced density matrix ${\rho }_{Q}$, describing the qubit only, is obtained by tracing over the environment degrees of freedom. The Lindblad master equation for the density matrix ${\rho }_{Q}$ is:

2 $$\frac{d{\rho }_{Q}(t)}{dt}=-\,i[H(t)+{H}_{LS}(t),{\rho }_{Q}(t)]+{{\mathscr{D}}}_{t}[{\rho }_{Q}(t)],$$

where *H*_*LS*_(*t*) is the Lamb shift Hamiltonian and ${{\mathscr{D}}}_{t}$ is the dissipator, responsible for the non unitary dynamics. They are both described in terms of local (in time) Lindblad operators *L*(*t*). A detailed description of *H*_*LS*_(*t*), ${{\mathscr{D}}}_{t}$ and the Lindblad operators is given in the Supplementary Information.

In this paper we focus on two “quality” estimators:

*The residual energy* ${\varepsilon }_{{\rm{res}}}$, which tells us whether our adiabatic dynamics is successful (or not) in reaching the target state and is defined as the difference between the energy of the system at the final time *t*_*f*_ and the exact ground state ${E}_{0}({t}_{f})$ of the target Hamiltonian $H({t}_{f})$.

3 $${\varepsilon }_{{\rm{res}}}=Tr[{\rho }_{Q}H({t}_{f})]-{E}_{0}({t}_{f}).$$

Of course, due to the adiabatic theorem^28^, *if the evolution is unitary*, ${\varepsilon }_{{\rm{res}}}$ tends to zero when ${t}_{f}\to \infty$.

*The Leggett*-*Garg*’*s correlation functions*, which tell us if the system behaves quantum coeherently during its dynamics^24^.

We focus on the third-order Leggett-Garg’s function, in terms of two times correlation functions *C*_*i*,*j*_:

4 $${K}_{3}^{a}={C}_{12}+{C}_{23}-{C}_{13}$$

5 $${C}_{i,j}=\langle {\sigma }_{z}({t}_{i}){\sigma }_{z}({t}_{j})\rangle $$

and other nonequivalent third order functions ${K}_{3}^{b}=-\,{C}_{12}-{C}_{23}-{C}_{13}$, and ${K}_{3}^{c}=-\,{C}_{12}+{C}_{13}+{C}_{13}$ obtained by nontrivial cycling of the 1, 2, 3 indexes (all the other permutations are trivially reduced to one of these three). If the system behaves classically, then $-3\le {K}_{3}^{i}\le 1$, $i\in \{a,b,c\}$. Hence, in the following, we seek for violation of Leggett-Garg’s inequalities during the annealing process, to make sure that our system behaves quantum mechanically up to the annealing time *t*_*f*_.

## Measurement scheme

Studying the LGI requires the evaluation of the two times correlation functions *C*_*i*,*j*_, which implies measuring the system twice during the annealing dynamics, as the configuration of the system is known at the initial time $s=0$. Conventional projective measurements^21^ are detrimental for the adiabatic quantum computation. Indeed after the measurement the system may populate instantaneously many excited states (see Supplementary Information) reducing the fidelity very close to zero. By contrast, we adopt the paradigm of weak measurements^29^, to gain relevant information from the LGI with negligible effect on the annealing results.

**Figure 1:**
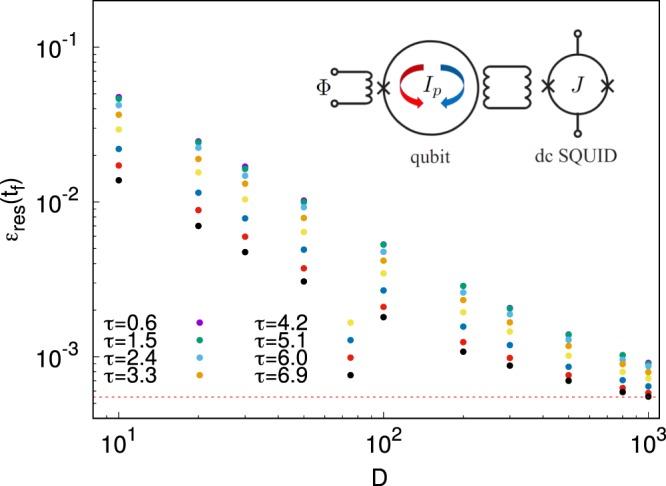
Log-log plot of the residual energy of the system at ${t}_{f}=14$ as a function of the variance *D* and of the times at which the measurements are performed: ${t}_{1}=\tau$ and ${t}_{2}=2\tau$. We observe that the residual energy decreases eventually going to $5.49\cdot {10}^{-4}$ which is the residual energy of the system in the absence of measurements for this choice of *t*_*f*_ (red dashed line at the bottom). The inset in the right top corner shows a sketch of the system-ancilla ensemble.

In particular we adopt the weak measurement approach designed in ref.^30^. The qubit described by a pseudospin degree of freedom *σ*_*z*_ is coupled to an ancilla device which is measured to extract information on the qubit state. The stronger the coupling between the two systems, the larger the invasiveness of the measurement on the quantum annealing dynamics. In order to specify an experimentally accessible weak measurement setup, following ref.^30^, we think of our system as a superconducting flux qubit, an RF SQUID for instance, which is inductively coupled to a hysteretic DC SQUID (the ancilla measuring apparatus), as shown in the inset of Fig. 1. At the measuring time, a short current pulse *I*_*b*_ biases the ancilla close but below its critical superconducting current *I*_*c*_. The duration of the pulse is one of the features charcterizing the so called discrimination time *T*_*V*_, for which the signal to noise ratio is close to unity^31^),The interaction between the two loops is given by ${H}_{I}=M{I}_{p}{\sigma }_{z}J$ where *I*_*p*_ is the current circulating in the qubit, *J*_*A*_ the current circulating in the ancilla and M the mutual inductance between the two. The expected direction of the current, clockwise or counterclockwise, influences the probability for the ancilla of switching or not switching to the dissipative state. The presence or absence of an output voltage at the ancilla SQUID is assumed as a measurement of the dicotomic value of the qubit observable *σ*_*z*_, which is intimately related to the qubit state. If the ancilla relaxation time *T*_*r*_ is shorter than *T*_*V*_, one cannot determine with certainty which is the output voltage. Let *x* be a continuous variable, associated to the switching or non switching of the ancilla at the time of the measurement. We may consider the value of *x* as binormally distributed, with peaks around the two values $x=\pm \,1$. We can assume the following probability distribution *P*(*x*, *t*)^32^:

6 $$P(x,t)={\rho }_{Q\downarrow \downarrow }(t)\,{P}_{-}(x)+{\rho }_{Q\uparrow \uparrow }(t)\,{P}_{+}(x),$$

where *P*_±_ are gaussians distributions with a variance $D\propto {T}_{r}/{T}_{V}$, centred around the two values $x=\pm \,1$ and reducing to *δ*–functions in the case of a projective measurement. The quantities ${\rho }_{Q\downarrow \downarrow }(t)$ and ${\rho }_{Q\uparrow \uparrow }(t)$ are the diagonal elements of the qubit density matrix ${\rho }_{Q}$ in the computational basis (↑, ↓) at time *t*. The density matrix of the qubit after the measurement, ${\rho ^{\prime} }_{Q}$, can be updated from the one before the measurement, ${\rho }_{Q}$, according to the rule^33,34^:

7 $${\rho ^{\prime} }_{Q}[x(t)]=\frac{1}{{\rho }_{Q\downarrow \downarrow }{e}^{\gamma }+{\rho }_{Q\uparrow \uparrow }{e}^{-\gamma }}(\begin{array}{cc}{\rho }_{Q\downarrow \downarrow }{e}^{\gamma } & {\rho }_{Q\downarrow \uparrow }\\ {\rho }_{Q\downarrow \uparrow }^{\ast } & {\rho }_{Q\uparrow \uparrow }{e}^{-\gamma }\end{array}),$$

where $\gamma =x(t)/D$. It is evident that the smaller is *D* (also, the shorter is the pulse), the less the system is perturbed.

The simulation implies that the value for *x* is extracted many times in order to obtain meaningful information on the qubit. each time the subsequent evolution of the system is determine by the extracted value for *x* according to Eq. (6). The average value for *x* obtained from the different results is used to calculate the correlation functions *C*_*ij*_.

This approach allows us to simultaneously measure spin-spin correlation functions, with negligible effect on the annealing dynamics, hence on the residual energy as it will be clearer in the following (detail on our weak measurement scheme can be found in the Supplementary Information).

## Single qubit annealing dynamics

In our model hamiltonian, we choose ${t}_{f}=14$ as annealing time. In the unitary limit, this guarantees that the adiabatic condition is fully satisfied. Adiabaticity is evident by analyzing the behaviour of the residual energy ${\varepsilon }_{res}$, defined in Eq. (3) for the annealing Hamiltonian of Eq. (1), as a function of $t/{t}_{f}$ at fixed ${t}_{f}=14$ and the corresponding ground state population ${\rho }_{Q\downarrow \downarrow }({t}_{f})$, qualified as the “fidelity” (see Supplementary Information Fig. S1 bottom left panel). At the final time the residual energy is very close to zero ${\varepsilon }_{res}=5.49\times {10}^{-4}$ and the fidelity is ${\rho }_{Q\downarrow \downarrow }({t}_{f})=0.999$, when no spin-spin correlation functions are “measured” during the annealing dynamics, in agreemeent with ref.^25^.

Next, we analyse how the measurements required to evaluate the LGI affect the annealing dynamics, when there is no coupling to the environment. As the Hamiltonian is time dependent, we expect that correlations *C*_*i*,*j*_ will depend on each of the two times $({t}_{i},{t}_{j})$ separately. However we will choose in the following three equally spaced times with $\tau ={t}_{2}-{t}_{1}={t}_{3}-{t}_{2}$ to build up ${K}_{3}^{i}$ of Eq. (5), because maximum violation is expected in this case, at least in the absence of a dissipative bath.

In this way, we perform two weak measurements per annealing and then calculate the expectation values *C*_*i*,*j*_ by averaging over $N={10}^{6}$ repeated dynamics. In Fig. 1 we show the residual energy, obtained for different choices of the variance *D*, at different values of $\tau$. The larger the variance *D*, the less the system is affected by the measurements and the closer is ${\varepsilon }_{res}$ to its unperturbed value at the chosen annealing time ${t}_{f}=14$, drawn as a dashed red line at the bottom for reference. ${\varepsilon }_{res}$ at $D=20$ ranges from $7.0\times {10}^{-3}$ to $2.5\times {10}^{-2}$ depending on $\tau$. Correspondingly, the fidelity ranges from 0.973 to 0.993.

**Figure 2:**
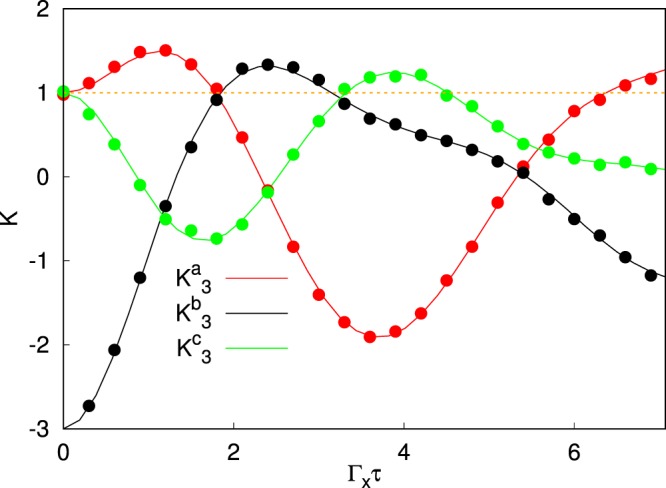
Plot of the Leggett-Garg’s function, in the absence of coupling to the environment. The lines are obtained performing projective measurements. The dots are calculated with weak measurements considering $N={10}^{6}$, $D=20$. ${K}_{3}^{a}$ in red, ${K}_{3}^{b}$ in black and ${K}_{3}^{c}$ in green. The orange line marks the bound of the LGI. Here $\tau ={t}_{2}-{t}_{1}={t}_{3}-{t}_{2}$, $\tau \in [0,{t}_{f}/2]$.

The choice of ${t}_{f}=14$ and $D=20$ is also satisfactory for the reliability of the measured correlations *C*_*i*,*j*_. In Fig. 2 we show the Leggett-Garg’s functions during the annealing dynamics in the unitary case, by comparing projective measurements with weak measurements at $D=20$. The bold lines are obtained in the case of projective measurements, while the dots represent the weak measurement results. They are hardly distinguishable. The agreement between projective and weak measurements in the evaluations of *C*_*i*,*j*_ demonstrate that our method reproduces the same correlations, at least in the unitary case. While in a single “weak” measurement procedure, occasional violations may be driven by the system-detector coupling, the average over $N={10}^{6}$ repeated measurements with $D=20$, assures the convergence to the projective results, with limited damage on ${\varepsilon }_{res}$ and ${\rho }_{Q\downarrow \downarrow }({t}_{f})$.

We clearly see that all the Leggett-Garg’s functions go beyond the “classical” limit, marked by an orange dashed line in Fig. 2. ${K}_{3}^{b}$ and ${K}_{3}^{c}$ are violated at intermediate times while ${K}_{3}^{a}$ for short and long times. Remarkably, at short times, ${K}_{3}^{a}$ and ${K}_{3}^{b}$ show complementary violations, as well as in the case of a single qubit oscillating between its two states, at its own frequency (without annealing)^24^. This is possibly due to the fact that, at short times, the qubit hamiltonian is “mostly” proportional to *σ*_*x*_^23^. Moreover, at $\tau ={t}_{f}/2=7$ there is a significant violation of ${K}_{3}^{a}$. This value corresponds to a final measurement time ${t}_{3}={t}_{f}$. Hence a violation of ${K}_{3}^{a}$ at $\tau ={t}_{f}/2$, is particularly relevant, because it is a direct proof of quantum coherence until the end of the annealing dynamics. Hence we will focus on the long time ($\tau \sim {t}_{f}/2$) behavior of ${K}_{3}^{a}$ in the following.

We are going to show now quantitatively how the coupling to a dissipative environment during the annealing process is disruptive for the quantum coherence. We have checked that, with the choice of ${t}_{f}=14$ and $D=20$, the convergence of the annealing procedure towards the target state is preserved also in this case and we stick to this choice in the rest of the simulations.

**Figure 3:**
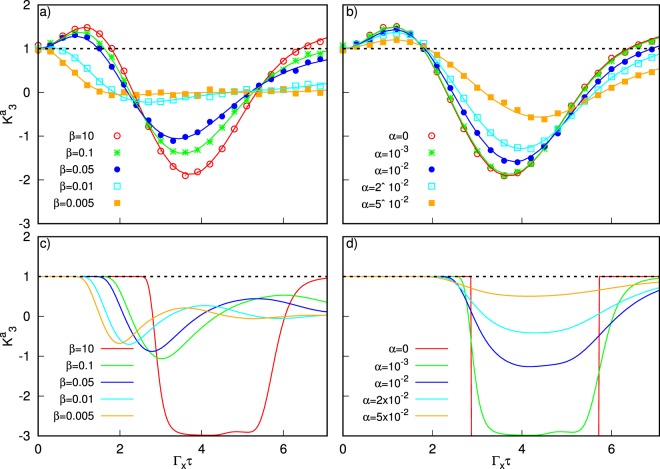
Plot of the Leggett-Garg’s function ${K}_{3}^{a}$ during the annealing dynamics. The black dashed line highlights the upper bound for the LGIs. The LG’s functions are plotted as a function of the difference of the times at which the measurements are perfomed: ${t}_{2}-{t}_{1}={t}_{3}-{t}_{2}=\tau$ (in units of $\hslash /{\Gamma }_{x}$ with $\hslash =1$). The time $\tau$ goes from 0 to *t*_*f*_/2 so that it scans the whole evolution (${t}_{f}=14$). Top panels present results of quantum simulations, bottom panels of classical Langevin dynamics described in the Supplementary Information. In (**a** and **c**) we set $\alpha ={10}^{-3}$, in (**b** and **d**) $\beta =10$.

Indeed, when turning on the dissipative interaction between the system and the bath (system-bath coupling $\alpha \ne 0$, cutoff frequency for the ohmic spectral density, ${\omega }_{c}=25$), the value of ${K}_{3}^{a}$ drops below the classical bound for the LGIs at the final time both by increasing the temperature (see Fig. 3a) and by increasing *α* (see Fig. 3b). However, we argue that conditions can be met by which a generic quantum computation can be successful, even during the LGI testing.

It is evident that the temperature plays a key role in the detrimental effect of the thermal bath. For low temperatures the quantum behavior persists during the whole evolution even in the presence a finite of coupling with the environment. By contrast, increasing the temperature, the time during which the system shows quantum features decreases, eventually going to zero for very high temperatures.

We have used a master equation in the Lindblad form to account for the dissipative environment, with the *caveat* that this approach guarantees reliable results only in the weak coupling limit. Therefore the results shown for very high temperatures, and strong coupling, might be beyond our approximation and have to be considered with caution. Moreover, at high temperatures, also the description of a SQUID flux-qubit as a two level system ceases to be correct. Therefore, we focus on results at low temperatures ($\beta =10$) and weak-intermediate couplings. In this case (Fig. 3b)), the ${K}_{3}^{a}$ function violates the unitary limit at long times all the range from $\alpha =0$ up to $\alpha ={10}^{-3}$.

However, by increasing the coupling up to $\alpha ={10}^{-2}$, the value of ${K}_{3}^{a}$ is very close to its “classical” upper bound. Hence, in this case, we decided to compare the quantum Lindblad dynamics with a classical Langevin dynamics of a corresponding analogous classical system, to assess the LG functions in the absence of quantum correlations (see Supplementary Information). Results are presented in the bottom panels of Fig. 3c,d. Interestingly enough, the high temperature results (see Fig. 3a,c) show close resemblance between classical and quantum dynamics. As expected, the LG functions never violates their classical upper bounds, though (dashed line). At intermediate couplings, quantum dynamics does not allow for full violation of LGI. Nontheless, the behavior of ${K}_{3}^{a}$ shows remarkable and qualitative differences with respect to the classical case. In particular we notice that the ordering of the curves as a function of the coupling at the annealing time corresponding to $\tau =7$ is reversed between the quantum and the classical case, except for the cases *α* = 0, 10^−3^. Hence, even in these borderline cases, the evaluation of LGI’s could be relevant to asses whether the dynamics has occurred via a quantum or a classical path. A comparative analysis of long time dynamics can be found in the Supplementary Information. Of course we cannot make strong claims comparing Langevin and Lindblad dynamics, but this correspondence points to poor quantum correlations in the Lindblad dynamics at higher couplings. In the case of low temperatures (see Fig. 3b,d), at weak coupling, we find a fully different behavior of ${K}_{3}^{a}$, which can be hence used as a ‘quantum estimator’.

## Generalization to many qubits

A generalization of our annealing process to many interacting qubits starts from the N spin 1/2 each represented by *σ*^(*k*)^, $k=1,\ldots N$)

8 $$H(s)=(1-s)\frac{{\Gamma }_{x}}{2}\,\mathop{\sum }\limits_{k}^{N}\,{\sigma }_{x}^{(k)}+s\frac{{\Gamma }_{z}}{2}\,\mathop{\sum }\limits_{k}^{N}\,{\sigma }_{z}^{(k)}-s\,\mathop{\sum }\limits_{k < \ell }^{N}\,{\bar{J}}_{k\ell }{\sigma }_{z}^{(k)}{\sigma }_{z}^{(\ell )}.$$

The interaction between the qubits ${\bar{J}}_{k\ell }$ is turned on at $t > 0$. We work in the computational basis, where each of the 2^*N*^ states spanning the Hilbert space, takes the form:

9 $$|{\psi }_{i}\rangle ={|{\sigma }_{z}^{(1)}{\sigma }_{z}^{(2)}\ldots {\sigma }_{z}^{(N)}\rangle }_{i}={|{\sigma }_{z}^{(1)}\rangle }_{i}\otimes {|{\sigma }_{z}^{(2)}\rangle }_{i}\otimes \ldots \otimes {|{\sigma }_{z}^{(N)}\rangle }_{i},$$

where $|{\sigma }_{z}^{k}{\rangle }_{i}$ is spin *S*_*z*_ of the *k*^*th*^ spin in the *i*^*th*^ many body state. The density matrix elements in this representation are

10 $${\rho }_{ij}{=}_{i}\langle {\sigma }_{z}^{(1)}{\sigma }_{z}^{(2)}\ldots {\sigma }_{z}^{(N)}|\hat{\rho }{|{\sigma }_{z}^{(1)}{\sigma }_{z}^{(2)}\ldots {\sigma }_{z}^{(N)}\rangle }_{j}=\langle {\psi }_{i}|\hat{\rho }|{\psi }_{j}\rangle .$$

Our goal is to evaluate the LGI during the annealing evolution by measuring just one of the qubits (let us say the first) while evolving (and annealing) the whole system. In this case, the generalization of the single qubit case to many qubits is easily done as shown in the following. This approach gives considerable information on the system in reasonable interaction strength regimes. However for large qubit ensembles, as already noted in ref.^35^, measuring the total spin of the system could allow for larger violations of LGI. It follows that the latter could be a smarter choice when choosing the dichotomic variable. However, although simultaneous measurements of many qubits have already been addressed^36,37^, their generalisation to a weak measurement approach, as the one described by us, has not been considered till now. It is far from trivial and is beyond the purpose of this paper.

In the following, our choice is to weakly measure the correlation functions *C*_*i*,*j*_ necessary to evaluate the Leggett-Garg functions just for the z-component of the first spin ${\sigma }_{z}^{(1)}$:

11 $${C}_{i,j}=\langle {\sigma }_{z}^{(1)}({t}_{i}){\sigma }_{z}^{(1)}({t}_{j})\rangle .$$

**Figure 4:**
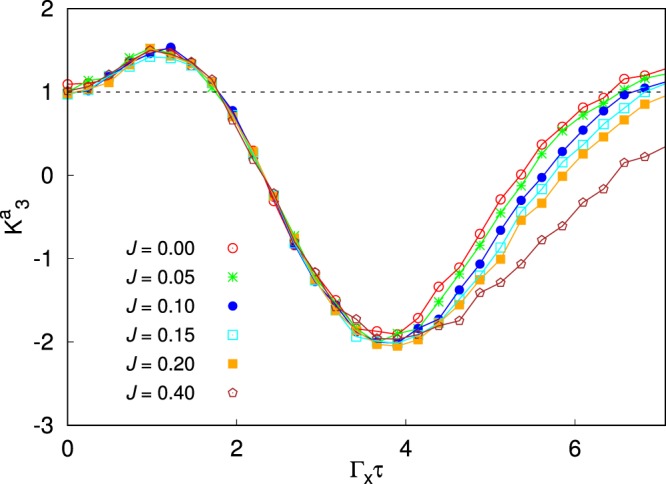
Plot of the Leggett-Garg’s function ${K}_{3}^{a}$ during the annealing dynamics in the absence of system-bath coupling for a two qubit system for increasing values of the exchange coupling $\bar{J}$ between them. The black dashed line highlights the upper bound for the LGIs. The LG’s functions are plotted as a function of the difference of the times at which the measurements are performed: ${t}_{2}-{t}_{1}={t}_{3}-{t}_{2}=\tau$. The time $\tau$ goes from 0 to *t*_*f*_/2 so that it scans the whole evolution (${t}_{f}=10\sqrt{2}$).

**Figure 5:**
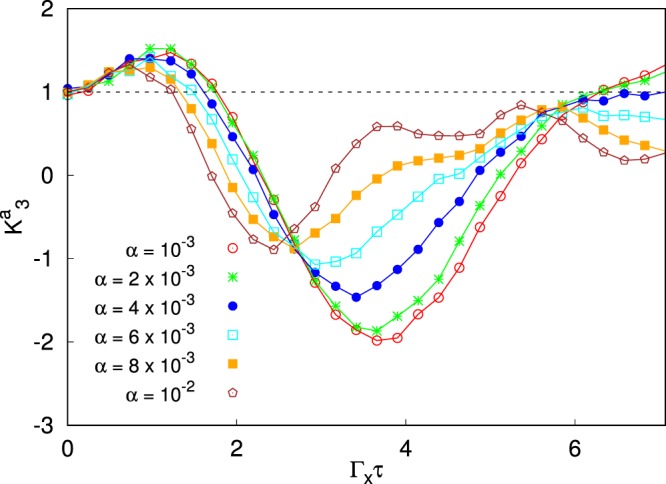
Plot of the Leggett-Garg’s function ${K}_{3}^{a}$ during the annealing dynamics in the presence of system-bath coupling of strength *α*, for a two qubit system with the exchange coupling between them $\bar{J}=0.2$ and inverse temperature $\beta =10$. The black dashed line highlights the upper bound for the LGIs. The LG’s functions are plotted as a function of the difference of the times at which the measurements are performed: ${t}_{2}-{t}_{1}={t}_{3}-{t}_{2}=\tau$). The time $\tau$ goes from 0 to *t*_*f*_/2 so that it scans the whole evolution (${t}_{f}=10\sqrt{2}$).

As only the first spin is coupled to the detector, Eq. (6) modifies as follows:

12 $$P(x,t)={P}_{-}(x)\,\sum _{i|{\sigma }_{z}^{(1)}=\downarrow }\,{\rho }_{ii}(t)+{P}_{+}(x)\,\sum _{i|{\sigma }_{z}^{(1)}=\uparrow }\,{\rho }_{ii}(t).$$

where ${P}_{\pm }(x)$ have been defined after Eq. (6). Here ${P}_{\pm }(x)$ are multiplied by the probability that the first spin is “measured” up or down, respectively. Correspondingly, the two sums are restricted to the diagonal density matrix elements for states having the first spin up or down, respectively. Hence, following the same line of reasoning of single qubit case (see Supplementary Information), we can work out the update scheme of the density matrix from $\rho ({t}_{ < })$, for *t*_<_ just before the measurement, to $\rho ^{\prime} [x({t}_{ > })]$, for *t*_>_ just after the weak measurement:

13 $$\begin{array}{rcl}{\rho ^{\prime} }_{ii}[x({t}_{ > })] & = & \{\begin{array}{l}\tfrac{1}{P(\gamma )}{\rho }_{ii}\,{e}^{\gamma },when\,first\,spin\,is\,measured\,^{\prime} \uparrow ^{\prime} \\ \tfrac{1}{P(\gamma )}{\rho }_{ii}\,{e}^{-\gamma },when\,first\,spin\,is\,measured\,^{\prime} \downarrow ^{\prime} \end{array},\\ {\rho ^{\prime} }_{ij}[x({t}_{ > })] & = & \tfrac{1}{P(\gamma )}{\rho }_{ij}{\rm{.}}\end{array}$$

(with $\gamma =x(t)/D$). In Fig. 4 we show a calculation of ${K}_{3}^{a}$, in the case of two qubits evolving according to Eq. (8), with ${\Gamma }_{x}={\Gamma }_{z}=1$, for increasing values of ${\bar{J}}_{12}={\bar{J}}_{21}=\bar{J}$ (in units of ${\Gamma }_{x}$) as a function of $\tau ={t}_{3}-{t}_{2}={t}_{2}-{t}_{1}$ in the absence of the coupling to the dissipative bath, performing weak measurements with $D=20$ and averaging over $N={10}^{6}$ measurements. Notice that at $\bar{J}=0$ one of the qubit is completely decoupled and is not involved in the measurement. Hence the curve at $\bar{J}=0$ can be used to compare two and single qubit dynamics. In order to highlight the potentiality of our technique, in Fig. 5, we stick to $\bar{J}=0.2$, and turn on the system-bath coupling *α*, at inverse temperature $\beta =10$. Even in the case of two qubits, our technique seems to be very promising. We find the maximal violation of ${K}_{3}^{a}({t}_{f}/2)$ at the final time $\tau ={t}_{f}/2$ for $\alpha =0$ gets smaller and smaller when increasing the system-bath coupling and eventually drops below the unitary limit for $\alpha  > 4\cdot {10}^{-3}$. The violation of LGI at the final time $\tau ={t}_{f}/2$ is also heavily reduced when increasing the exchange coupling $\bar{J}$ between the two spins, even in the absence of system-bath coupling, as reported in Fig. 4. For a strongly interacting system ($\bar{J} > 0.4$), the violation of LGI no longer occurs. Such a lack of violation has to be ascribed to the choice of the observable ${\sigma }_{z}^{(1)}$ as the observable to be monitored, by introducing the corresponding dichotomic variable *x*. This choice does not grasp the complexity of a fully interacting system. Choosing an appropriate observable to be measured, which maximizes the LGI violation at long times is a very relevant issue and will be the subject of further investigation.

## Discussion

Quantum Annealing is in principle a successful technique to reach the target state at the annealing time. However, the question whether the dynamics has driven the system through a quantum or a classical path, has been only partially addressed in the literature and has remained unanswered up to now. The method proposed here to assess the quantumness of a system during its adiabatic evolution is based on the LGIs that we evaluate in the framework of weak measurements. It appears as powerful and experimentally accessible. The time correlations can be measured without perturbing the annealing dynamics. The LGIs hold information about the interaction with the environment and can be used as witness of quantum coherence. In this work we have provided a quantitative estimate of the detrimental effect of repeated measurements during the QA evolution, in the presence of a dissipative environment, by studying the quantum coherence of two coupled two level systems. The thermal bath has been modelized within the Lindblad scheme, which is reliable at small couplings. Our findings confirm that conditions can be met under which the QA procedure may keep an amount of quantum correlations over the full QA evolution, even when measuring to check the LGIs.

Do our results allow us to answer the point raised at the beginning of this paper? Namely: are real annealers, claimed to work performing adiabatic quantum computation, really quantum annealers? Are their outcomes macroscopic manifestations of quantum mechanics?

Our results show, for a very simple model, that if one measures the LGIs along the adiabatic dynamics, a possible, yet non trivial, outcome could be that ${K}_{3}^{a}({t}_{f}/2)$ contains all the information we need, to assess if the final result of the computation is quantum or not. In the case of a long time violation ${K}_{3}^{a}({t}_{f}/2) > 1$, the result has to be considered as quantum, even in the presence of a dissipative bath. In borderline cases ${K}_{3}^{a}({t}_{f}/2)\sim 1$, a careful analysis of the ${K}_{3}^{a}$ behaviour as a function of time, could unveil the characteristics of the dynamics, namely if it was quantum, classical, or due to a non trivial occurrence of quantum and classical mechanisms. In this context, some of us have also shown that, under special circumstances, thermal activation may also improve annealing performances^38,39^. However this approach is still at its infancy. We have highlighted the fact that the result is crucially bound to the choice of the observable to be measured, when monitoring the survival of those quantum correlations that are considered to be the relevant ones in the specific many-body system under study. In the case of strongly interacting many-qubits systems, the total spin of the system has been proven to give maximal violations for the LGI^35^. It would be of great interest extending our rationale to different measurement procedures so that also a simultaneous measurement of multiple qubits is allowed.

## Supplementary information

Supplementary Material

## Data Availability

The data that support the plots within this paper and other findings of this study are available from the corresponding author upon request.
