# Supplementary material for: Assessing the quantumness of the annealing dynamics via Leggett Garg’s inequalities: a weak measurement approach
Source: Sci Rep. 2019 Sep 20;9:13624. doi: 10.1038/s41598-019-50081-8 (PMC6754466; doi:10.1038/s41598-019-50081-8)
Supplement: Supplementary file 1 — Supplementary Material [file 41598_2019_50081_MOESM1_ESM.pdf]

Assessing the quantumness of the annealing dynamics via Leggett Garg's inequalities: a weak measurement approach

V. Vitale[1], G. De Filippis[1,2], A. De Candia[1], A. Tagliacozzo[1,2], V. Cataudella[1,2], and P. Lucignano[2,1]

[1]Dipartimento di Fisica "Ettore Pancini", Università di Napoli "Federico II", Monte S. Angelo, I-80126 Napoli, Italy
[2]CNR-SPIN, Monte S. Angelo via Cinthia, I-80126 Napoli, Italy
[*]procolo.lucignano@spin.cnr.it

1 Leggett-Garg's Inequalities

The starting point of the Leggett-Garg's Inequalities is a definition of macrorealism as a principle one wants to stick at ("is the flux there when nobody looks?"). This is contained in a small set of principles or assumptions that, quoting directly from[1], reads:

A: **Macroscopic realism per se**. A macroscopic object which has available to it two or more macroscopically distinct states is, at any given time, in a definite one of those states.

B: **Non-invasive measurability**. It is possible in principle to determine which of these states the system is in, without any effect on the state itself or on the subsequent system dynamics.

C: **Induction**. The properties of ensembles are determined exclusively by initial conditions (and in particular not by final conditions).

These properties define what has been called "classicity" or "macrorealism".
Based on the assumptions above, Leggett and Garg derived Bell's-like inequalities that any system behaving classically should obey[2]. Violations of these inequalities provide evidence of quantum behavior of a system if one accepts that the alternative to classical probabilities is quantum mechanics. Therefore these violations can be interpreted as an indicator of the "quantumness" of a system.

Following Ref.[3], in this section we briefly introduce the Leggett-Garg's inequalities and discuss their properties as witness of "quantumness".
Let us begin with the definition of a classical dichotomic variable Q which can assume value +1 or -1: $Q(t_i) = Q_i$ stands for the measurement value of the observable at time t_i. We denote with $P_i(Q_i)$ the probability of obtaining the result Q_i at time t_i. The correlation function $C_{i,j}$ can be defined as follows:

$$C_{i,j} = \sum_{Q_i,Q_j=+,-1} Q_i Q_j P_{ij}(Q_i,Q_j) = \langle Q_i Q_j \rangle, \tag{1}$$

where the subscripts of P remind us of the times at which the measurements were performed. Assumption A, that is "Macrorealism per se", guarantees that P_{ij} can be obtained as the marginal probability of $P_{ijk}(Q_i,Q_j,Q_k)$.

$$P_{ij}(Q_i,Q_j) = \sum_{Q_k;t_k\neq t_i,t_j} P_{ijk}(Q_i,Q_j,Q_k) \tag{2}$$

The assumption of "Non-invasive measurability" allows to drop the subscripts of P_{ijk} and use the $P(Q_3,Q_2,Q_1)$ alone (with $\sum_{Q_3,Q_2,Q_1} P(Q_3,Q_2,Q_1) = 1$) to calculate the three correlation functions: $C_{1,2}, C_{2,3}, C_{1,3}$. We obtain

$$\begin{aligned}
C_{1,2} &= P(+,+,+) + P(+,+,-) - P(-,+,-) + P(-,-,+) + P(-,-,-) - P(+,-,+) - P(+,-,-) - P(-,+,+),\\
C_{1,3} &= P(+,+,+) + P(+,-,+) - P(-,-,+) + P(-,+,-) + P(-,-,-) - P(+,+,-) - P(+,-,-) - P(-,+,+),\\
C_{2,3} &= P(+,+,+) + P(-,+,+) - P(-,-,+) + P(+,-,-) + P(-,-,-) - P(+,+,-) - P(-,+,+) - P(+,-,+),
\end{aligned} \tag{3}$$

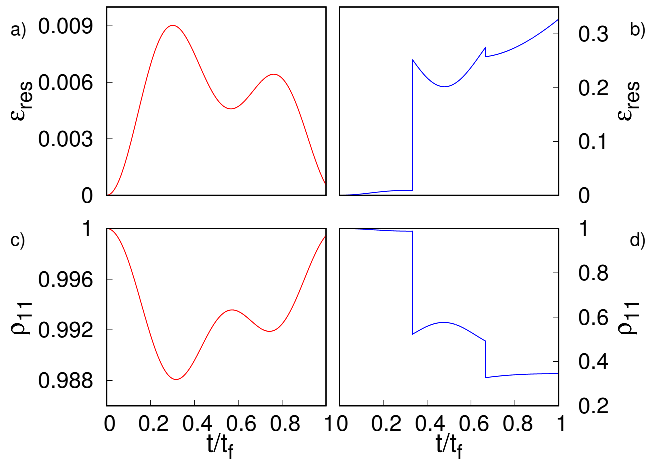

Figure 1. S

Residual Energy and ground state population (Fidelity) for unitary annealing evolution as a function of t/t_f ($t_f = 10\sqrt{2}$). a) Residual Energy in the absence of measurements; b) Residual Energy in the case of projective measurements at times $t_2 = 0.3\,t/t_f$ and $t_3 = 0.6\,t/t_f$; c) Fidelity in absence of measurements; d) Fidelity in the case of measurements at times $t_2 = 0.3\,t/t_f$ and $t_3 = 0.6\,t/t_f$

where $\pm$ stands for $Q = \pm$. Next, we define

$$K_3^a = C_{1,2} + C_{2,3} - C_{1,3} = 1 - 4[P(+,-,+) + P(-,+,-)]. \tag{4}$$

The upper bound of K_3^a corresponds to $P(+,-,+) = P(-,+,-) = 0$, giving $K_3^a = 1$; the lower bound, instead, $K_3^a \geq -3$ corresponds to $P(+-,+) + P(-,+,-) = 1$. Besides the inequality

$$-3 \leq K_3 \leq 1 \tag{5}$$

other inequalities exist, that can be found in the literature.
Various symmetry properties can be used to derive other constrains on the correlations. The change $Q \to -Q$ in K_3^a gives rise to the following inequality:

$$-3 \leq K_3^b \leq 1; \quad K_3^b \equiv -C_{1,2} - C_{2,3} - C_{1,3}. \tag{6}$$

Finally, the last, different, third order inequality can be obtained from K_3^a, just changing a sign:

$$-3 \leq K_3^c \leq 1; \quad K_3^c \equiv -C_{1,2} + C_{2,3} + C_{1,3}. \tag{7}$$

These are the only three different inequalities that can be derived from correlations to third order. Higher order inequalities can also be constructed.

2 Measurement scheme

In this Section we sketch an idealized measurement approach which can be extended from projective to weak measurement, to reduce the invasiveness of the classical measurement process. Resorting to a weak measurement scheme is unavoidable to allow for a successful annealing. Indeed in Fig. 1S we show the residual energy and the ground state population during the annealing dynamics in the absence and in the presence of two projective measurements in order to demonstrate the necessity of weakening the measurement approach. In the panels a) and c) we show the residual energy and the ground state population in the absence of measurements. At the annealing time the latter is approximately 1 and the former is nearly 0 which reveals that the quantum annealing has been successful. On the other hand, in the panels b) and d) we calculate the residual energy and the ground state population, while measuring one of the possible $C_{2,3}$ necessary to build the K_3s. Clearly, at the measurement times t_2 and t_3, the (projective) measurement procedure suddenly alters the population of the ground state. The ground state population is very poor at the annealing time, and the residual energy considerably larger that zero, signalling that the annealing procedure has failed. Thus approaching to the calculation of the Leggett-Garg's functions with weak measurements is necessary

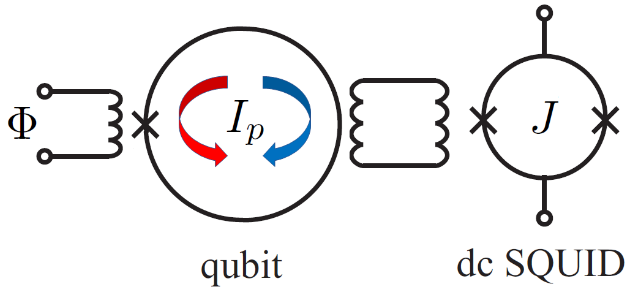

Figure 2. S
Diagram of the superconducting flux qubit coupled to the hysteretic DC SQUID.

to evaluate the coherence of the system during the quantum annealing with negligible effects on the dynamics.
For the sake of concreteness, we consider a superconducting flux qubit, as the system S, and an hysteretic DC SQUID, as the ancilla/detector (Fig.2S). In a superconductiong flux qubit, when polarized by an external flux ϕ close to $\phi_0/2$, where ϕ_0 is the flux quantum, the current can flow clockwise or anti-clockwise. A spin degree of freedom can be associated to the circulating current, e.g. the state of the system can be denoted by $|\uparrow\rangle$, if the current is clockwise, while it is $|\downarrow\rangle$, if the current is anti-clockwise.
On this basis of eigenstates of σ_z, the Hamiltonian of the flux qubit can be written as in the main text:

$$H_S = \frac{\Gamma_x}{2}\sigma_x + \frac{\Gamma_z}{2}\sigma_z, \tag{8}$$

where Γ_x is the tunnel coupling between the current states and $\Gamma_z = 2I_p(\frac{\phi_0}{2} - \phi)$, where I_p is the magnitude of the current flowing in the flux qubit.
By introducing a linear time dependence in the same Hamiltonian one obtains:

$$H(s) = (1-s)\frac{\Gamma_x}{2}\sigma_x + s\frac{\Gamma_z}{2}\sigma_z, \tag{9}$$

which is the Hamiltonian that describes a linear annealing protocol. Here $s = t/t_f$ goes from 0 to 1 and t_f is the total annealing time. The system evolves along its dynamics and, to evaluate the possible violation of the Leggett-Garg's inequalities, it is necessary to measure correlations of system variables at different times. The quantum state of the system can be read out by exploiting the mutual inductance M of the qubit with the ancilla DC SQUID, represented by the interaction Hamiltonian $H_I = MJI_p\sigma_z$. Here J is the current circulating in the DC SQUID.
As described in ref.[4], the measurement can be performed considering that the ancilla, appropriately polarized with J close to the critical current I_c, by means of a flux $\Phi \sim n\phi_0$ (n integer), can be either in a superconducting state with zero voltage $V = 0$ or in a dissipative state with a finite voltage $\bar{V}$. Let the circulating current in the ancilla be $J < I_c$, prior to measurement. With a short current pulse, the DC SQUID can be biased very close to its critical current I_c. During the pulse the ancilla has a certain probability of staying in the $V = 0$ state, or switching to the dissipative state depending on the state of the qubit $|\uparrow\rangle, |\downarrow\rangle$. Indeed, the circulating current I_p induces an electromotive force in the ancilla loop, which increases or decreases I_b, its bias current, respectively. After the pulse, I_b is maintained stable at a value $I_b = I_c/2$ while the ancilla relaxes in one of its two possible states in order to measure the voltage output. If the relaxation time of the ancilla T_r is much larger than the so-called discrimination time T_V one can obtain meaningful information from a measurement and infer the qubit state[5]. If T_r is not long enough compared to T_V, such that a single measurement cannot provide the full information to evaluate the ancilla voltage and, consequently, the current state of flux qubit, the measurement becomes minimally invasive and weakly perturbs the quantum coherence of the evolution. An estimate of T_V, can be given by requiring that the signal-to-noise ratio approaches unity . This occurs when the spectral density $S_V(f)$ of the output noise of the detector at frequency f can be approximated as

$$S_V(f) = \lim_{\tau_m \to \infty} \frac{2\,|V_{RMS}(f)|^2}{\tau_m} \approx \frac{2\,|V_{RMS}(f)|^2}{T_V}. \tag{10}$$

To be more specific, let us map the values $V = 0$ and $V = \bar{V}$ onto a dimensionless variable x which assumes values ± 1: $V = \bar{V}(1+x)/2$. The probability $P(x)$, of reading a value x after the measurement is

$$P(x,t) = \rho_{Q\downarrow\downarrow}(t)\,P_-(x) + \rho_{Q\uparrow\uparrow}(t)\,P_+(x). \tag{11}$$

where ρ_Q is the reduced density matrix of the flux qubit given in Eq.(30) and $P_\pm$ is the probability of having a value of $x=\pm$, as the result of the measurement. $P_\pm(x)$ can be viewed as two gaussian distributions centered around $x=\pm1$ with the variance $D=\frac{T_r}{T_V}$ in analogy with Ref.[6] where a conceptually similar approach is investigated. The change of the time of measurement in the experiment amounts to tuning the width of the $P_\pm(x)$ peaks. The Ansatz of a Gaussian distribution is due to the fact that a long measurement process gives $V=0$ or $V=\bar{V}$ with probability $\rho_{Q\downarrow\downarrow}$ or $\rho_{Q\uparrow\uparrow}$, while, by taking a short interaction time between qubit and ancilla, strange voltage values are not excluded[7]. Repeating the experiment many times, a bimodal distribution is expected with two unequal peaks centered at $V=0$ or $V=\bar{V}$, respectively. Of course, no matter how weak the measurement is, the density matrix ρ_Q of the system turns out to be slightly modified, depending on the outcome of the measurement of the variable x. Following Ref.[8], the transformation from ρ_Q (before the measurement) to ρ'_Q (after the measurement) is defined as (time label omitted):

$$\begin{aligned} \rho'_{Q\downarrow\downarrow} &= \frac{\rho_{Q\downarrow\downarrow}\,P_-(x)}{P(x)} \\ \rho'_{Q\downarrow\uparrow} &= \rho_{Q\downarrow\uparrow}\sqrt{\frac{\rho'_{Q\downarrow\downarrow}\rho'_{Q\uparrow\uparrow}}{\rho_{Q\downarrow\downarrow}\rho_{Q\uparrow\uparrow}}}, \quad \rho'_{Q\uparrow\downarrow} = \rho'^{*}_{Q\downarrow\uparrow} \\ \rho'_{Q\uparrow\uparrow} &= \frac{\rho_{Q\uparrow\uparrow}\,P_+(x)}{P(x)}. \end{aligned} \tag{12}$$

This expression can be written in a more convenient form. From Eq.(12) we get

$$\frac{\rho'_{Q\downarrow\downarrow}}{\rho'_{Q\uparrow\uparrow}} = \frac{\rho_{Q\downarrow\downarrow}}{\rho_{Q\uparrow\uparrow}}\frac{P_-(x)}{P_+(x)} = \frac{\rho_{Q\downarrow\downarrow}}{\rho_{Q\uparrow\uparrow}}\,e^{\frac{2x}{D}}. \tag{13}$$

Let us denote with γ the x/D ratio and get

$$\rho'_{Q\downarrow\downarrow}\rho_{Q\uparrow\uparrow}e^{-\gamma} = \rho'_{Q\uparrow\uparrow}\rho_{Q\downarrow\downarrow}e^{\gamma}, \tag{14}$$

then

$$\rho'_{Q\downarrow\downarrow} = \frac{\rho_{Q\downarrow\downarrow}\,e^{\gamma}}{\rho_{Q\downarrow\downarrow}\,e^{\gamma}+\rho_{Q\uparrow\uparrow}\,e^{-\gamma}}. \tag{15}$$

Therefore, one obtains the following quantum-map from ρ to ρ':

$$\rho'_Q = \frac{1}{\rho_{Q\downarrow\downarrow}e^{\gamma}+\rho_{Q\uparrow\uparrow}e^{-\gamma}}\begin{pmatrix}\rho_{Q\downarrow\downarrow}\,e^{\gamma} & \rho_{Q\downarrow\uparrow} \\ \rho^{*}_{Q\downarrow\uparrow} & \rho_{Q\uparrow\uparrow}\,e^{-\gamma}\end{pmatrix} \tag{16}$$

The value of x is stored for evaluating the correlation functions C_{ij}.

By tuning D, we are able to weaken the measurement till the post-measurement update in the density matrix is negligible. This is crucial if the goal is of looking at the Leggett-Garg's correlations during an annealing dynamics, without spoiling the quantum coherence of the qubit too much.

To sum up, the annealing protocol that we have realized in the simulation is the following. Firstly, one prepares the system in the ground state of the Hamiltonian $H(0)$. The system evolves under $U=e^{-iH(t/t_f)t}$. Computationally this means solving the differential equation for the density matrix Eq.(30) with a fourth-order Runge-Kutta algorithm. At fixed times t_1,t_2(or t_2,t_3 or t_1,t_3) one performs two weak measurements, by extracting values of x, which are used to evaluate C_{ij} and to update the density matrix at the corresponding times by means of the probability distribution of Eq.(11).

To gain sufficient statistics, the same evolution is repeated up to 10^6 times and the Leggett-Garg's correlation functions are evaluated as an average on the different runs.

In this way, the Leggett-Garg's inequalities can be tested with weak measurements with minimal perturbation of the system during its dynamics.

The idealized measurement approach described here hides a number of experimental challenges. For a study on the back action of the detector on the flux qubit, on the problems related to the Joule heating in the dissipative state and on the fidelity of the weakness of the measurement we refer to Ref.[5] and references therein.

3 Lindblad approach to the quantum dissipative environment

In this Section we recall the quantum dynamics of an open quantum system described by the time dependent Hamiltonian $H_S([A_\alpha],t)$, interacting with an ohmic thermal bath in the weak coupling Lindblad approach. $[A_\alpha]$ are a set of Hermitian and

dimensionless operators of the system while the bath is described by the Hermitian Hamiltonian $H_B([B_v])$, where $[B_v]$ are operator describing the bath. Let U_S and U_B be the time evolution operators of the system and bath only:

$$U_S(t,t') = \mathscr{T}\, \mathrm{e}^{-\mathrm{i}\int_{t'}^{t} H_S(\tau)\mathrm{d}\tau}; \tag{17}$$

$$U_B(t,t') = \mathrm{e}^{-\mathrm{i}H_B\left(t-t'\right)}. \tag{18}$$

Here $\mathscr{T}$ is the time ordering operator in real time. The evolution of the system and the bath in the absence of interaction is governed by

$$U_0(t,t') = U_S(t,t') \otimes U_B(t,t'). \tag{19}$$

The interaction between system and bath is described, in full generality, by

$$H_\mathrm{I} = \sum_\alpha g_\alpha A_\alpha \otimes B_\alpha, \tag{20}$$

where g_α are coupling constants. Adiabatic switching of the interaction at negative times is assumed. We define the total Hamiltonian for the joint system-bath universe,

$$H(t) = H_S(t) + H_B + H_I, \tag{21}$$

and the time-dependent density operator $\rho(t)$, whose dynamics is expressed by the Von Neumann equation and the full system-bath evolution operator,

$$U(t,t') = \mathscr{T}\, \mathrm{e}^{-\mathrm{i}\int_{t'}^{t} H(\tau)\mathrm{d}\tau}. \tag{22}$$

Moving to the interaction picture, we define

$$\tilde{U}(t,0) = U_0^\dagger(t,0)U(t,0), \tag{23a}$$

$$\tilde{\rho}(t) = U_0^\dagger(t,0)\rho(t)U_0(t,0), \tag{23b}$$

$$\tilde{H}_I(t) = U_0^\dagger(t,0)H_I(t)U_0(t,0), \tag{23c}$$

where

$$\widetilde{H}_\mathrm{I}(t) = U_0^\dagger(t,0)H_\mathrm{I}U_0(t,0) = g\sum_\alpha A_\alpha(t) \otimes B_\alpha(t), \tag{24}$$

and $A_\alpha(t)$, $B_\alpha(t)$ are the time-evolved operators,

$$A_\alpha(t) = U_S^\dagger(t,0)A_\alpha U_S(t,0), \tag{25}$$

$$B_\alpha(t) = U_B^\dagger(t,0)B_\alpha U_B(t,0). \tag{26}$$

$\tilde{U}(t,0)$ and $\tilde{\rho}(t)$ satisfy the following differential equations:

$$\begin{cases} \frac{d}{dt}\tilde{U}(t,0) = -i\tilde{H}_I(t)\tilde{U}(t,0), \\ \tilde{U}(0,0) = \mathbb{I}, \end{cases} \tag{27a}$$

$$\begin{cases} \frac{d}{dt}\tilde{\rho}(t) = -i[\tilde{H}_I(t),\tilde{\rho}(t)], \\ \tilde{\rho}(0) = \mathbb{I}. \end{cases} \tag{27b}$$

Given the two point correlation function $\mathscr{B}_{\alpha\beta}(\tau) \equiv \left\langle B_\alpha(\tau)B_\beta(0)\right\rangle$, the spectral-density matrix of the bath is:

$$\Gamma_{\alpha\beta}(\omega) \equiv \int_0^\infty d\tau\, e^{i\omega\tau}\mathscr{B}_{\alpha\beta}(\tau) = \frac{1}{2}\gamma_{\alpha\beta}(\omega) + iS_{\alpha\beta}(\omega), \tag{28}$$

where itsreal and imaginary part are

$$\begin{aligned} \gamma_{\alpha\beta}(\omega) &= \int_{-\infty}^{\infty} d\tau e^{i\omega\tau}\mathscr{B}_{\alpha\beta}(\tau) = \gamma^*_{\alpha\beta}(\omega), \\ S_{\alpha\beta}(\omega) &= \int_{-\infty}^{\infty} \frac{d\omega'}{2\pi}\gamma_{\alpha\beta}(\omega')P(\frac{1}{\omega-\omega'}) = S^*_{\alpha\beta}(\omega). \end{aligned} \tag{29}$$

Lindblad theory eventually leads to master equation for the reduced density matrix (representing only the system variables),

$$\frac{\mathrm{d}\rho_Q(t)}{\mathrm{d}t} = -\mathrm{i}\left[H(t)+H_{\mathrm{LS}}(t),\rho(t)\right]+\mathscr{D}\left[\rho_Q(t)\right], \tag{30}$$

where the adiabatic dissipator $\mathscr{D}$ is

$$\mathscr{D}\left[\rho_Q(t)\right] = \sum_{\alpha\beta}\sum_{\omega}\gamma_{\alpha\beta}(\omega)\left[L_{\beta\omega}(t)\rho_Q(t)L^{\dagger}_{\alpha\omega}(t)-\frac{1}{2}\left\{L^{\dagger}_{\alpha\omega}(t)L_{\beta\omega}(t),\rho_Q(t)\right\}\right] \tag{31}$$

and the Lamb shift Hamiltonian takes the form

$$H_{\mathrm{LS}}(t) = \sum_{\alpha\beta}\sum_{\omega}S_{\alpha\beta}(\omega)L^{\dagger}_{\alpha\omega}(t)L_{\beta\omega}(t). \tag{32}$$

They are written in terms of the Lindblad operators $L_{\alpha\omega}(t)$, which are defined as

$$L_{\alpha\omega}(t) = \sum_{\varepsilon_a(t)-\varepsilon_b(t)=\omega}\left|\varepsilon_a(t)\right\rangle\left\langle\varepsilon_a(t)\middle|A_\alpha\middle|\varepsilon_\beta(t)\right\rangle\left\langle\varepsilon_b(t)\right|, \tag{33}$$

where $\{\,\varepsilon_a(t)\,\}$ are the instantaneous eigenvectors of the system Hamiltonian.

The universe we study here is a single spin coupled to a bath of bosonic harmonic oscillators described by the Hamiltonian

$$H_B = \sum_{k=1}^{\infty}\omega_k b^{\dagger}_k b_k, \tag{34}$$

where $b^{\dagger}_k$ and b_k are, respectively raising and lowering operators for the k-th oscillator with frequency ω_k and the frequency spectrum is assumed to be continuous as usually in the spin-boson model[9–11]. The bath is assumed to be in thermal equilibrium at inverse temperature $\beta = 1/k_B T$, so that Its density operator is just $\rho_B = \mathrm{e}^{-\beta H_B}/\mathscr{Z}$.

The interaction between the system and the bath is $H_I = \sigma_z \otimes B$, where the operator B is defined $B = \sum_k g_k(b^{\dagger}_k + b_k)$. The Fourier transform of the bath correlation function is:

$$\gamma(\omega) = \frac{2\pi J\left(|\omega|\right)}{1-\mathrm{e}^{-\beta|\omega|}}g^2\left(\Theta(\omega)+\mathrm{e}^{-\beta|\omega|}\,\Theta(-\omega)\right), \tag{35}$$

where $\Theta(\pm\omega)$ are Heaviside functions[9]. The model is fully determined once the explicit form of the function $J(\omega)$ is given. In this paper, we consider an Ohmic bath[10], characterized by

$$J(\omega) = \eta\,\frac{\omega^{\nu}}{\omega_{\mathrm{c}}^{\nu-1}}\,\mathrm{e}^{-\omega/\omega_{\mathrm{c}}}, \quad \text{with } \nu = 1, \tag{36}$$

where ω_{c} is a high-frequency cut-off that is the maximum phonon energy and η is a dimensional parameter with dimensions of time squared. ω_{c} has been chosen $25\,\Gamma_x$ in the simulations. In conclusion, we define $\alpha = \eta g^2$ and explicit γ as follows:

$$\gamma(\omega) = 2\pi\alpha\frac{\omega\, e^{-\frac{|\omega|}{\omega_c}}}{1-e^{-\beta\omega}}, \tag{37}$$

4 Simulation of a two-level-system dissipative dynamics with classical Langevin dynamics

In this Section we consider the flux x of a flux qubit as a classical variable and we study its classical thermal evolution in a time dependent double well potential $U(x,t)$ that mimicks the superconducting flux qubit evolution. Such double well potentials have been used in the analysis of experimental realization of flux qubits[12]. By setting up a classical model evolution which corresponds to the single qubit evolution studied in the main text, we are able to obtain the LG functions for the classical system and highlight the close correspondence between the classical and the quantum outcome, when the quantum evolution provides LG functions very close to the classical limit.

Let us start defining the potential $U(x,t)$ at $t = 0$. The time independent Hamiltonian of the flux variable is given by:

$$\hat{H} = -\frac{\hbar^2}{2m}\frac{\partial^2}{\partial x^2}+U(x), \tag{38}$$

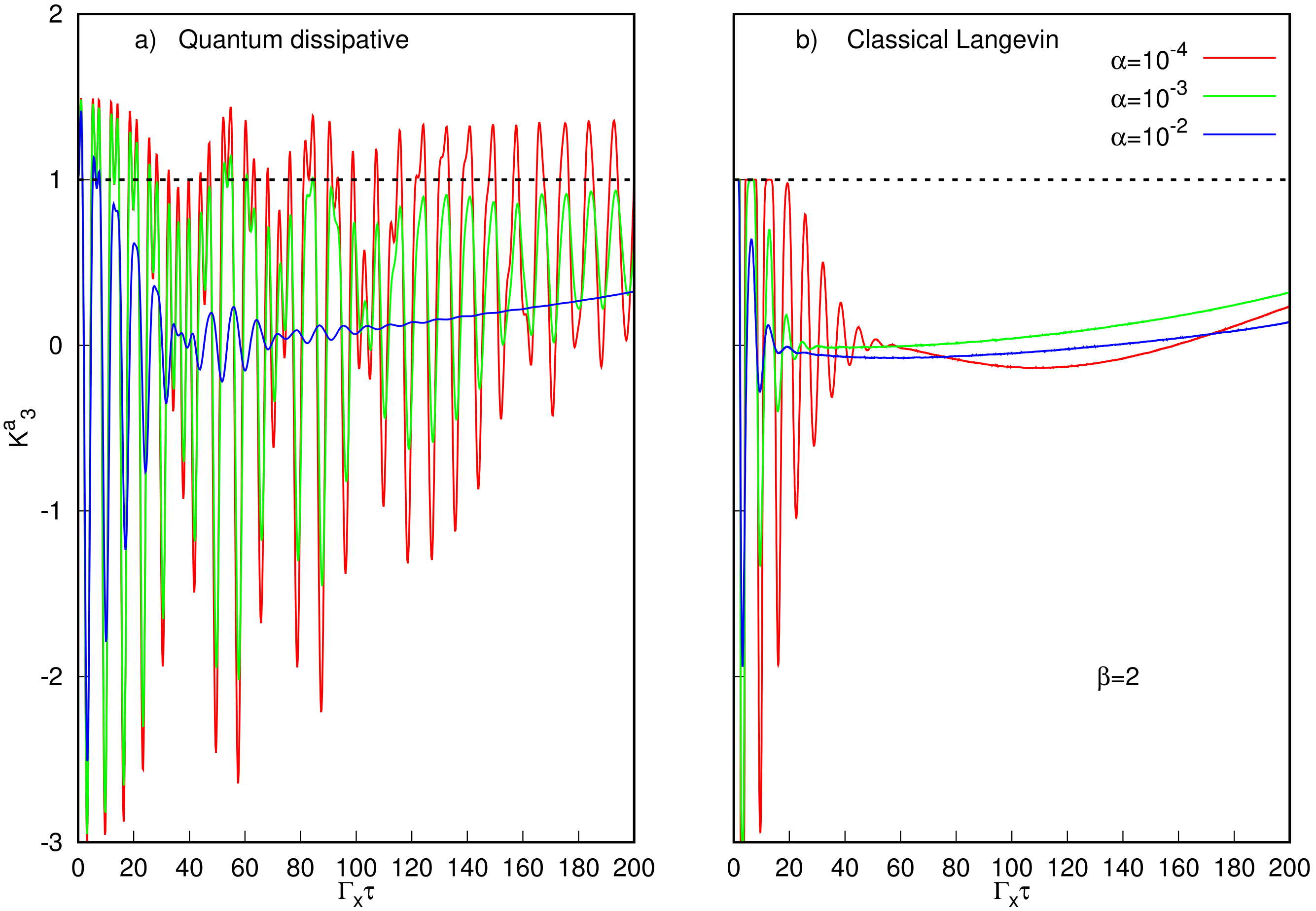

Figure 3. S
Plot of the Leggett-Garg's function K_3^a during the annealing dynamics for very long annealing time $t_f = 400$. The black dashed line highlights the upper bound for the LGIs. The LG's functions are plotted as a function of the difference of the times at which the measurements are performed: $t_2 - t_1 = t_3 - t_2 = \tau$ in units of Γ_x. The time τ goes from 0 to $t_f/2$ so that it scans the whole evolution ($t_f = 400$). The two panels present results of quantum simulations, (panel a), and of classical Langevin dynamics (panel b). The inverse temperature is $\beta = 2$.

where the mass is typically related to the device capacitance and the potential has two minima. The lowest energy wavefunctions of the potential, have to correspond to the two states $|\uparrow\rangle$ and $|\downarrow\rangle$ of the qubit of Eq. (8). Parameters Γ_x and Γ_z are given respectively by

$$\Gamma_x = 2\,\langle\downarrow|\hat{H}|\uparrow\rangle, \tag{39a}$$

$$\Gamma_z = \langle\downarrow|\hat{H}|\downarrow\rangle - \langle\uparrow|\hat{H}|\uparrow\rangle. \tag{39b}$$

The case $\Gamma_z = 0$ corresponds to a symmetrical double well, while $\Gamma_x = 0$ corresponds to a very high potential barrier, so that the states do not overlap and the matrix element between them vanishes.

To construct the potential at $t = 0$, we first approximate the potential $U(x)$ near the two minima with two harmonic wells. Next, we consider the lowest energy wavefunctions centered in each of the two harmonic wells and the corresponding states $|L\rangle$ and $|R\rangle$. The two wave functions always overlap to some extent, as long as the potential barrier between the wells in $U(x)$ is finite and their wavefunctions have to be orthogonalized.

The states $|\uparrow\rangle$ and $|\downarrow\rangle$ are superpositions of $|L\rangle$ and $|R\rangle$ with wavefunctions in which the variable x is mostly confined in each of the two wells of the potential.

A generic time dependent double well potential takes the form:

$$U(x,t) = U_0 \left[\frac{1}{4}x^4 - \frac{1}{2}a(t)x^2 - h(t)x\right], \tag{40}$$

where U_0 and the functions $a(t)$ and $h(t)$ are chosen to simulate the desired time evolution. Note that the potential has the two required minima as long as $a(t)^3 > \frac{27}{4}h(t)^2$. The states with the lowest energy eigenvalues are:

$$|\downarrow\rangle = p\,|L\rangle + q\,|R\rangle\,, \tag{41a}$$

$$|\uparrow\rangle = p\,|R\rangle + q\,|L\rangle\,, \tag{41b}$$

where $p(t)$ and $q(t)$ are fixed by the condition that the norms are unitary and the scalar product is zero. We then impose the constraints determined by Eqs.(39), where $\Gamma_x(t)$ and $\Gamma_z(t)$ are given functions of time. In particular, we choose a linear annealing procedure where $\Gamma_x(t) = \Gamma_0\left(1 - \frac{t}{t_f}\right)$, $\Gamma_z(t) = \Gamma_0\frac{t}{t_f}$. Here Γ_0 is a parameter with the dimension of an energy. At time $t = 0$, the double well is symmetrical, so that $h(0) = 0$. We also set $a(0) = x_0^2$, so that the two minima at time zero are in $\pm x_0$. Note that setting the values of Γ_0 and x_0 amounts to setting the units of energy and length. For definiteness, we take $\Gamma_0 = h\nu_0$, with $\nu_0 = 1$ GHz. Therefore, at time $t = 0$ we have two free parameters, U_0 and the mass m of the particle, and only one equation, Eq. (39a), to satisfy. Eq. (39b) is satisfied already, being $h(0) = 0$. This leaves one free parameter, that we fix by imposing that the quantum ground state of the potential is higher than the barrier, and the classical oscillation frequency of the particle inside the well is equal to the Rabi frequency of the quantum two level system, that is ~ 1 GHz. For times $t > 0$, we leave U_0 and m constant, and fix $a(t)$ and $h(t)$ so that the desired annealing schedule of $\Gamma_x(t)$ and $\Gamma_z(t)$ is realized. With time, the height of the barrier grows in order to make $\Gamma_x(t)$ decrease, becoming much higher than the ground states of the two wells, until at time $t = t_f$ it becomes virtually infinite (in practice we only require that $\Gamma_x(t_f)$ is 1/1000 of the initial value, to avoid dealing with an infinite barrier).
To study the "classical analogue" of the qubit dynamics, we have simulated the classical Langevin equation

$$\ddot{x} = -\frac{1}{m}\frac{\partial U(x,t)}{\partial x} - \int_0^t \gamma(t-t')\dot{x}(t') + \xi(t), \tag{42}$$

where $\xi(t)$ is a thermal noise with $\langle\xi(t)\rangle = 0$ and $\langle\xi(t)\xi(t')\rangle = \frac{k_B T}{m}\gamma(t-t')$. We fix the function $\gamma(t)$ postulating that Eq.(42) results from the same interaction with a thermal bath of harmonic oscillators as the spin σ_z, with x/x_0 playing the role of σ_z. This provides the interaction Hamiltonian with harmonic oscillators with frequency ω_k:

$$H_I = \frac{x}{x_0}\sum_k g_k(b_k^\dagger + b_k) + \left(\frac{x}{x_0}\right)^2 \sum_k \frac{g_k^2}{\hbar\omega_k}, \tag{43}$$

where b_k and $b_k^\dagger$ are destruction and creation operators of the oscillators, and g_k are coupling constants with the dimensions of an energy. The second term (the so called counter-term) ensures that the interaction with the bath does not modify the potential $U(x,t)$ of the particle It is a constant when $x/x_0 \to \pm 1$. Eliminating the bath degrees of freedom[13], we obtain that the variable x obeys Eq.(42) with

$$\gamma(t) = \frac{2}{\hbar m x_0^2}\int_0^\infty d\omega\,\frac{J(\omega)}{\omega}\cos\omega t, \tag{44}$$

where $J(\omega) = \sum_k g_k^2\,\delta(\omega - \omega_k)$. For an ohmic bath, consistently with Eq. (36), we set $J(\omega) = \alpha\omega e^{-\omega/\omega_c}$, so that the function $\gamma(t)$ in the limit of large ω_c becomes the delta function $\gamma(t) = \alpha\left(\frac{2\pi}{\hbar m x_0^2}\right)\delta(t)$.
Comparison of classical and quantum (dissipative) dynamics are shown in Fig. 3S , where we show the behaviour of the LG K_3^a functions for long annealing times. A detailed discussion of the short time dynamics can be found in the main paper. In the case of the long time dynamics shown in Fig. 3S, despite certain similarities, for instance in the oscillating period, the scales of the LG function decay appear to be fully different. In the quantum system, notwithstanding the dissipative environment, the damping of the K_3^a oscillations is much slower than in the classical case for all the dissipation strengths chosen.

References

1. Leggett, A. J. Testing the limits of quantum mechanics: motivation, state of play, prospects. *J. Physics: Condens. Matter* **14**, R415 (2002).

2. Leggett, A. J. & Garg, A. Quantum mechanics versus macroscopic realism: Is the flux there when nobody looks? *Phys. Rev. Lett.* **54**, 857–860, DOI: 10.1103/PhysRevLett.54.857 (1985).

3. Emary, C., Lambert, N. & Nori, F. Leggett–garg inequalities. *Reports on Prog. Phys.* **77**, 016001 (2014).

4. Picot, T., Schouten, R., Harmans, C. & Mooij, J. Quantum nondemolition measurement of a superconducting qubit in the weakly projective regime. *Phys. review letters* **105**, 040506 (2010).

5. Lupaşcu, A., Verwijs, C., Schouten, R., Harmans, C. & Mooij, J. Nondestructive readout for a superconducting flux qubit. *Phys. review letters* **93**, 177006 (2004).

6. Williams, N. S. & Jordan, A. N. Weak values and the leggett-garg inequality in solid-state qubits. *Phys. Rev. Lett.* **100**, 026804, DOI: 10.1103/PhysRevLett.100.026804 (2008).

7. Aharonov, Y., Albert, D. Z. & Vaidman, L. How the result of a measurement of a component of the spin of a spin-1/2 particle can turn out to be 100. *Phys. Rev. Lett.* **60**, 1351–1354, DOI: 10.1103/PhysRevLett.60.1351 (1988).

8. Jordan, A. N., Korotkov, A. N. & Büttiker, M. Leggett-garg inequality with a kicked quantum pump. *Phys. Rev. Lett.* **97**, 026805, DOI: 10.1103/PhysRevLett.97.026805 (2006).

9. Albash, T., Boixo, S., Lidar, D. A. & Zanardi, P. Quantum adiabatic markovian master equations. *New J. Phys.* **14**, 123016 (2012).

10. Breuer, H. P. & Petruccione, F. *The Theory of Open Quantum Systems* (OUP Oxford, 2007).

11. Caldeira, A. O. & Leggett, A. J. Quantum tunnelling in a dissipative system. *Annals Phys.* **149**, 374 (1983).

12. Harris, R. *et al.* Quantum annealing with manufactured spins. *Nature* **473**, 194–198 (2011).

13. Weiss, U. *Quantum Dissipative Systems* (World Scientific, 2012).
